# International health experiences in postgraduate medical education: A meta-analysis of their effect on graduates’ clinical practice among underserved populations Expériences internationales de soins de santé en formation médicale post-doctorale : une méta-analyse des effets sur la pratique clinique des diplômés au sein des populations mal desservies.

**DOI:** 10.36834/cmej.56940

**Published:** 2020-08-06

**Authors:** Russell Dawe, Mark McKelvie

**Affiliations:** 1 Memorial University of Newfoundland, Newfoundland and Labrador, Canada; 2 Queen’s University, Ontario, Canada

## Abstract

**Background:**

International health experiences (IHEs) are popular among medical learners and provide a valuable learning experience. IHE participants have demonstrated an increased intention to care for underserved populations in the future, but what is its actual impact on practice? This study evaluates the effect of postgraduate IHE participation on the future careers of clinicians regarding their work among underserved populations.

**Methods:**

We conducted a systematic review and meta-analysis of peer-reviewed articles comparing the populations served by physicians who had participated in an IHE with those of physicians who had not participated in an IHE.

**Results:**

764 titles were scanned, 28 articles were reviewed, with an eventual 3 studies of fair-good or good quality identified. These addressed physicians’ service to domestic underserved populations, and also addressed future service in a low- or middle-income country (LMIC). Meta-analysis demonstrated a statistically-significant increase in service by IHE graduates to domestic underserved populations (OR = 2.12; CI = 95%; P = 0.03). The certainty of the evidence was low due to limitations in study design (non-randomised studies) and inconsistency in effects.

**Conclusion:**

Participation in an IHE may cause an increase in care for domestic underserved populations in future clinical practice, though further research from high quality randomised trials is needed to increase the certainty of the effect. Further study is needed to establish whether there is a similar effect with increased future service in a LMIC setting.

## Abstract (fr)

**Contexte:**

Les expériences internationales de soins de santé sont populaires auprès des étudiants en médecine et fournissent une expérience d’apprentissage enrichissante. Les participants aux expériences internationales de soins de santé démontrent une intention accrue de prodiguer des soins aux populations mal desservies dans leur future pratique,; mais quelle est l’incidence réelle sur la pratique ? Cette étude évalue l’effet de la participation des résidents aux expériences internationales de soins de santé sur leur carrière future au sein des populations mal desservies.

**Méthodes:**

Nous avons réalisé une revue systématique et une méta-analyse des articles évalués par des pairs comparant les populations desservies par les médecins ayant participé aux expériences internationales de soins de santé et celles desservies par des médecins n’ayant pas participé à celles-ci

**Résultats:**

764 titres ont été analysés et 28 articles ont été révisés pour arriver à identifier trois études de qualité passable à bonne. Ces études portaient sur les services médicaux aux populations locales mal desservies, et de la pratique future dans un pays à revenu faible ou intermédiaire. La méta-analyse a démontré une augmentation statistiquement significative des soins aux populations locales mal desservies fournis par les diplômés avant participé à des expériences internationales de soins de santé (RC = 2,12; IC = 95 %; valeur p = 0,03). Le degré de certitude des preuves était plutôt bas en raison des limites associées aux devis (études non randomisées) et des tailles d’effet inconstantes.

**Conclusions:**

La participation aux expériences internationales de soins de santé peut augmenter les soins médicaux aux populations locales mal desservies dans une future pratique clinique, mais des recherches supplémentaires à partir d’essais randomisés de haute qualité doivent être menées pour établir la certitude de l’effet. D’autres études sont nécessaires pour savoir s’il existe un effet similaire lié à l’augmentation de la pratique future dans un pays à revenu faible ou intermédiaire.

## Introduction

International health experiences (IHEs) are common among undergraduate and postgraduate medical education programs. As early as 1996, a survey found that 192 (43%) of 442 North American family medicine residency programs either offered an IHE of their own or encouraged residents to participate in an IHE otherwise.^1^ In 2000, over 38% of North American medical school graduates surveyed had taken part in an IHE.^1^ In 2010, a survey identified 80 global health fellowships in 7 specialties across the US.^2^ This market demand for IHEs has led many institutions in the US to increasingly invest in such training experiences for medical students and residents.^3^ However, there is growing evidence to show that facilitating IHEs may do more than respond to learner interests.

Many authors have suggested that domestic health care systems benefit from physicians who have received part of their training internationally in a low- or middle-income country (LMIC).^4^^-^^8^ For example, Greysen et al found that out of 521 physicians surveyed with formal training in clinical and health services research and policy leadership, 44% had some global health experience during or after their training, and 85% of respondents perceived that their global health activities had improved the quality of their work back home.^3^ These physicians reported that IHEs inspired increased work among vulnerable populations, advocacy, and research on the social determinants of health.

IHEs can be resource-intensive^9^ and government or other institutional sources of funding often expect a domestic health return on their financial or human resource investment.^10^ It is not intuitive for stakeholders to believe that sending a trainee away on an IHE will result in increased service to local underserved populations. However, multiple studies demonstrate the positive impact of IHEs on medical learners’ intention to care for domestic underserved populations.^11^^,^^12^ For example, among US medical students who had participated in a 2-year international health fellowship, 80% (35/44) reported an intention to practice “primarily in the United States and from time to time overseas.”^13^ Greysen found that among their 229 respondents who identified having had any global health (GH) experience during or after their training, 73% indicated that <10% of their total professional time was dedicated to international health work in the past year.^3^

If IHEs are found to increase physicians’ care for domestic underserved populations, then funding such training could prove to be a strategic investment. However, much of the information gathered to date has been surveys of learners’ intentions to practice. Less is known about the actual practice patterns of clinicians after they have entered clinical practice.^3^ The purpose of this systematic review and meta-analysis is to evaluate the level of evidence indicating which populations physicians serve after completing postgraduate (i.e., any level of residency, including fellowship) training with some IHE component. What is the effect of participation in a postgraduate IHE upon graduates’ future practice among domestic and/or international underserved populations?

## Methods

### Eligibility criteria

We conducted a systematic review and meta-analysis of peer-reviewed articles which compared the populations served by physicians who had participated in an IHE with those of physicians who had not participated in an IHE. Types of IHEs vary significantly. To mitigate heterogeneity between programs studied we limited our search to IHEs occurring at the postgraduate level. Our inclusion criteria were: studies of physicians in clinical practice who had graduated from any residency or fellowship program with an IHE which involved some amount of time spent in a LMIC; outcomes reported must include location, socio-economic status (e.g., use of public assistance), or other indicator for population being served by the physician; a control group must be included; English-language, peer-reviewed articles. Exclusion criteria: intention-to-serve outcomes; undergraduate IHE experiences only; domestic-only IHE experiences (e.g., refugee health); non-English, non-peer-reviewed articles; abstracts. Peer-reviewed articles and abstracts were reviewed, but ultimately only full articles were accepted. One relevant abstract was identified, but after communication with the author, this was also excluded.

### Information sources

On April 24, 2018 we searched the following databases: PubMed, Cochrane, EMBASE, CINAHL, and Scopus; followed by hand-searching of reference lists among the relevant articles identified. Our methods for data collection and analysis followed the Preferred Reporting Items for Systematic Reviews and Meta-Analyses (PRISMA) checklist.^14^

### Search

Keywords and Medical Subject Headings (MESH) searched include: (((((“global health”[tiab] OR “international health”[tiab] OR underserved[tiab])) OR (((“Global Health/education” [Mesh]) OR “International Educational Exchange” [Mesh]) OR “Medically Underserved Area”[Mesh]))) AND (((career[tiab] OR careers[tiab] OR “career choice”[tiab] OR “career choices”[tiab] OR “practice location”[tiab] OR “postresidency”[tiab])) OR ((“Career Choice”[Mesh]) OR “Professional Practice Location”[Mesh]))) AND ((((“Internship and Residency”[Mesh]) OR “Education, Medical, Graduate”[Mesh])) OR (resident[tiab] OR residents[tiab] OR intern[tiab] OR interns[tiab] OR internship[tiab] OR graduate[tiab] OR graduates[tiab])). We did not set a search limit for date of publication. We did set a search limit for English-language only. After removing duplicate records, a total of 764 titles were identified.

### Study selection

**Figure 1:**
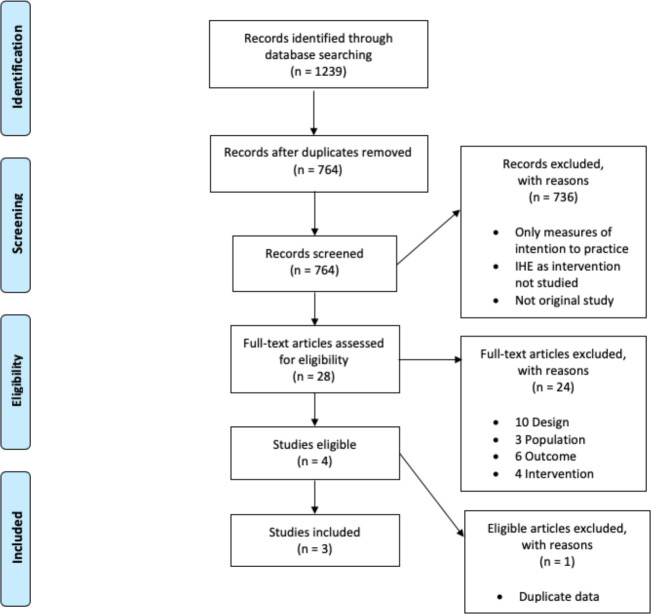
PRISMA flow diagram of study selection process.

Both authors independently scanned all 764 titles, and any title identified by either author was included for abstract review. Ninety-four abstracts were then reviewed independently by both authors, and any article identified by either author was then selected for full review. Twenty-eight full articles were reviewed by both authors independently, and consensus was achieved by discussion until both authors agreed upon which articles (*n* = 3) to include in the review and meta-analysis. See Figure 1 for PRISMA flow diagram.

### Data items and extraction

Two reviewers extracted information from all included studies pertaining to study characteristics, population, intervention and outcomes (Table 1). Study characteristics included country, sample size, and design and the population information was related to the type of residency program. Intervention information included the type of IHE (e.g., isolated elective, longitudinal track), the frequency of IHEs throughout residency, the duration of each IHE, and whether there was any associated global health curriculum taught in addition to the IHE itself. The outcomes of interest were number/proportion of physicians practicing among underserved patients in their domestic (non-LMIC) country and number/proportion of physicians practising internationally among underserved patients in a LMIC

**Table 1:** Description of studies included.

Study / Country	Design Data Source (Year)	Program Type, # Programs	Sample Size (Total, per group)	International Health Experience Description	Outcome Assessment Methods	Findings
**Liaw et al, 2014** USA	Retrospective cohort National Database* (1980-2009)	Family medicine 5 residency programs Universities not stated	n=999 graduates Control n=825 Intervention n=174	Exposure includes: global health elective or track. Total duration varied from 2-20 weeks divided among 1-3 trips over 4 years. Additional global health teaching provided: Yes	Location of practice in health professional shortage areas; medically underserved areas or populations; rural areas; areas of dense poverty; and any rural or underserved area.	68% (118/174) of GHE/GHT participants worked in any area of underservice vs. 60% (497/824) of nonparticipants (P=0.06). Not significant after logistic regression adjusts for confounders.
**Bazemore et al, 2011** USA	Retrospective cohort Participant Survey (1989-2003)	Family Medicine 1 residency program University of Cincinnati	n= 137 graduates Control n=52 Intervention n=43	Exposure includes: international health track. Total duration and frequency not stated. Additional global health teaching provided: Yes	Self-reported community volunteering and/or practice in underserved populations, rural areas, or developing nations during the first 5 years after residency.	59% (20/34) of IHT participants worked extensively with underserved populations vs. 42% (15/35) of non-participants post-IHT implementation (P=0.028).
**Gupta et al, 1999** USA	Retrospective cohort Participant Survey (1982-1996)	Internal Medicine 1 residency program Yale Medical School	n= 192 graduates Control n=52 Intervention n=96	Exposure includes: international health program. Total duration varied from 4-8weeks, frequency not stated. Additional global health teaching provided: not stated.	Self-reported clinical practice patient demographics, including patients on public assistance, immigrants, patients who are substance abusers, and patients infected with HIV.	80.2% (77/96) of IHP participants had >20% of their patients on social assistance vs. 51% (49/96) of non-participants (P<0.001). Other outcomes included: 43.8% (42/96) of IHP participants had substance abusers as >20% of their patients vs. 21.9% (21/96) of non-participants (P=0.001). 42.7% (41/96) of IHP participants had immigrants as >20% of their patients vs. 24% (23/96) of non-participants (P=0.006). 31.3% (30/96) of IHP participants had HIV patients as >20% of their patients vs. 13.5% (13/96) (P=0.003)

* American Medical Association Masterfile

### Quality assessment

Each author independently used the US Preventive Services Task Force (USPSTF) quality rating criteria^17^ to evaluate the quality of each article included and consider the risk of bias in each individual study. The authors then discussed their assessments and consensus was achieved, with the result that three studies were deemed good quality and one study, fair-good. See Table 2 for Assessment of studies using USPSTF quality grading criteria.

**Table 2:** Assessment of studies included in analysis using the US Preventive Services Task Force quality rating criteria.

Study	Groups Assembly	Groups Maintenance	Loss to follow-up	Measurements	Clear Intervention	Outcomes Considered	Analysis	Overall
Liaw, 2014	Good	Good	Good	Good	Good	Good	Good	Good
Bazemore, 2011	Good	Good	Good	Fair	Good	Good	Good	Good
Gupta, 1999	Good	Fair	Fair-Good	Fair	Good	Good	Good	Fair-Good

### Data synthesis

Meta-analysis was performed using Review Manager, version 5.3, comparing the impact of IHEs on population served. A Mantel-Haenzel odds ratio (OR) was calculated for each individual study and the results were combined to provide a pooled estimate of effect; OR with 95% confidence-intervals (CIs).

Since our research question was to provide an overall effect of any type of IHE for any type of residency program we anticipated there may be heterogeneity among the studies due to type of residency program, frequency and duration of the IHE and possibly in how the outcomes were assessed. To account for the possibility that these study differences may impact the effect we used a random effects model for the meta-analysis. No research ethics board approval was sought or required for this meta-analysis.

To provide further context and an assessment of the overall quality of the pooled estimate of effect, we used the GRADE approach to summarise and interpret the data. We created a summary of findings table for our two outcomes: number/proportion of physicians practicing among underserved patients in their domestic country (defined as non-LMIC); number/proportion of physicians practising internationally among underserved patients in LMIC (see Table 3). We used the five GRADE considerations (study design limitations, consistency of effect, imprecision, indirectness, and risk of bias) to assess the certainty of the evidence as it relates to the main outcomes.^18^ We used methods and recommendations described in Section 8.5 and Chapter 12 of the *Cochrane Handbook for Systematic Reviews of Interventions*.^19^

## Results

**Figure 2:**
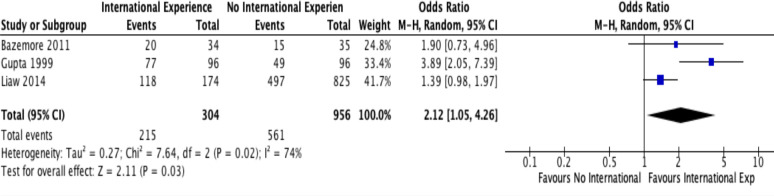
Forest plot assessing effect of IHE participation on future practice.

Of the 746 articles identified, three articles were found to meet the eligibility criteria regarding assessment of physicians’ service to a domestic underserved population and were included in this meta-analysis. All studies were conducted in the US and ranged in sample sizes from 137 to 999Two studies included residency programs in Family Medicine and one study included residency programs in Internal Medicine. Although two of the studies lacked a significant effect individually, when they were combined in our meta-analysis they demonstrated a statistically-significant positive effect overall (OR = 2.12; CI=95%; P=0.03). See Figure 2. Only one article meeting our inclusion criteria addressed service to LMICs, so this could not be assessed by meta-analysis. Two of the articles had outcomes for service to an overarching category of domestic underserved population (e.g., “medically underserved areas”). In these cases, this broad category, rather than any sub-categories within it, was used to perform the meta-analysis. One article had multiple overlapping categories of populations (e.g., “patients on public assistance” and “immigrant patients”), with no combined total. In this case, the category “patients on public assistance” was used, as this was believed to be the closest option to an overarching domestic underserved category available and also had the largest sample size to allow for a more precise estimate. See Table 1 for a description of each study included.

**Figure 3:**
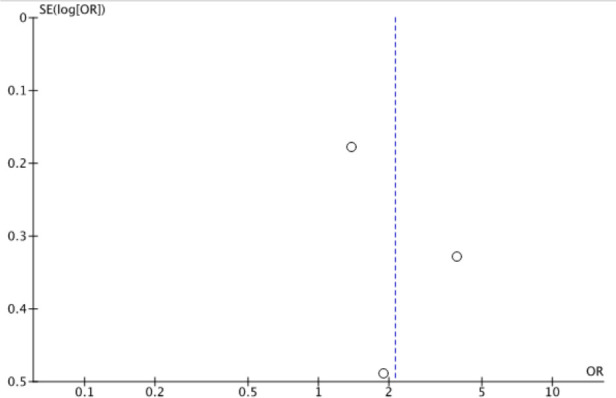
Funnel plot of included studies.

A high level of heterogeneity (I^2^ = 74%) was noted, so we used the random effects model rather than the fixed effects model. This heterogeneity may be due to the variability among different residency programs’ IHEs and/or differences in defining the outcome variable and measurement tool used. We created a funnel plot to assess for publication bias (see Figure 3); however, this is difficult to interpret with only 3 studies. A visual inspection of the funnel plot shows no strong evidence of publication bias, but studies with negative results could be lacking based on the absence of studies in the lower left end portion of the plot. The limited number of published studies makes this difficult to assess.

***Domestic service to underserved populations:*** Of the three studies with 1260 participants, we found a low certainty of evidence that the odds of providing medical care to domestic underserved populations as a result of participation in postgraduate medical programs with an IHE is 2.12 (95% CI: 1.05,4.26) times higher compared to participation in graduate residency programs without global health placements. The certainty was downgraded due to study design (as only retrospective cohort were used to assess effect) and inconsistency. See Table 3 for GRADE assessment of level of certainty of evidence.

**Table 3:** GRADE assessment of level of certainty of evidence.

Question: What is the effect of postgraduate international health experiences on physicians’ future practice among domestic and/or international underserved populations?			
**P:** practicing physicians who have graduated from residency **I:** participation in international health experience during residency **C:** no participation in international health experience during residency			
Outcome	OR (95% CI)	Participants (# of studies)	Certainty of evidence (GRADE)
Domestic service to underserved	2.12(1.05,4.26)	1260(3)	Low^1,4^ (+) (+) ( ) ( )
International service to LMIC	not estimable	69(1)	not estimable
Reason for downgrading: ^1^Study design limitations, ^2^Risk of bias, ^3^Imprecision, ^4^Inconsistency, ^5^Indirectness			

***International service to LMIC*:** We found one study (Bazemore) that also assessed IHE participants’ future provision of care in an LMIC. However, there was insufficient data reported to assess effectiveness on this outcome. See Table 3

### Effect of IHE on outcomes

***Domestic service to underserved populations*:** Of the three studies with 1260 participants, we found a low certainty of evidence that the odds of providing medical care to domestic underserved populations as a result of participation in postgraduate medical programs with an IHE is 2.12 (95% CI: 1.05,4.26) times higher compared to participation in graduate residency programs without global health placements. The certainty was downgraded due to study design (as only retrospective cohort were used to assess effect) and inconsistency. See Table 3 for GRADE assessment of level of certainty of evidence.

***International service to LMIC:*** We found one study (Bazemore) that also assessed IHE participants’ future provision of care in an LMIC. However, there was insufficient data reported to assess effectiveness on this outcome. See Table 3.

## Discussion

There is a broad range of structure and content in IHEs^1,^^8^ and the quality varies among them.^20^ Our study excluded undergraduate IHEs, as these have been previously reviewed elsewhere^20^ but did not otherwise restrict the type of IHE included. Our high level of heterogeneity, therefore, may be reflective of variable duration, content, or organizational structure among different IHEs. The question has been raised of which program characteristics determine whether an IHE will impact the residents’ future practice.^21^ For example, financial support, the presence of an established partner host site, adequate supervision while away, and mentorship at the home site, are key factors influencing residents’ choice to participate in international health electives.^22^ Such program characteristics may impact the quality of learning experience residents receive as well as which types of residents the program attracts, both of which may play a role in determining an IHE’s impact on future practice. Russ et al found that cumulative time spent on IHEs was strongly associated with future work in underserved or global health settings, more so than availability of mentorship or established partner host site.^23^ This suggests that duration of IHE would especially benefit from further investigation. However, given the limited number of published studies meeting our inclusion criteria, we were unable to conduct meaningful sub-group analyses for different characteristics of IHEs.

The authors of our three included studies acknowledge the risk of selection bias, commonly raised in this research. It is possible that residents who choose to participate in an IHE have a predisposition to work among the underserved. Therefore, studies with more rigorous design will be necessary before we can establish a definitive cause and effect relationship. Among these three studies, only Liaw et al.^16^ performed a logistic regression analysis to adjust for certain potential confounders among their IHE participants, such as minority status, which is known to increase likelihood of practice among the underserved.^25^ However, other potential confounders (e.g., rural birth) remain unaccounted for.^15^

Despite this potential for selection bias, residents’ pre-existing career plans may not be the main factor motivating most residents to participate in an IHE. Russ et al found that 11 out of 42 pediatric residents in an IHE program had previously planned to include global health in their future career.^23^ Additionally, Castillo et al found that 43% of pediatric residents agreed that their plans for global health to play an important role in their career was a factor in decision-making regarding their participation in an IHE.^22^ This placed plans for a career in global health as 10^th^ among 15 possible motivating factors. This shows that a small majority of IHE participants did not initially intend to pursue a career in global health. When comparing residents with previous IHE experience to those approaching an IHE for the first time, Castillo et al found that residents’ plans for a career involving global health was an influencing factor for 62% of IHE-experienced residents but only 27% of IHE-naïve residents (*P* < .0001).^22^ In short, residents with a history of multiple IHE experiences are more likely to have intentions to pursue careers in global health, whereas residents approaching their first IHE are most likely doing so for other reasons. Future studies which adjust for potential confounders should consider pre-existing interest in a career in an underserved or global health setting. Number of IHEs or cumulative time spent in previous IHEs (e.g., in or before undergraduate studies) may be a quantifiable marker useful for this purpose when evaluating the impact of postgraduate IHEs.

While the reasons remain unknown, from the three studies in this area, IHEs appear to have a positive effect on increasing graduates’ future clinical care for underserved populations. Also, the presence of an IHE in undergraduate and postgraduate medical education has been well-documented as an effective recruitment tool for the medical school or residency program in question.^1^^,^^12^^,^^25^ It is possible, therefore, that recruiting postgraduates who participate in IHEs may increase local service to underserved populations in that area. The healthcare system, academy, and community may therefore benefit as a result, regardless of whether IHEs instill a new social responsibility into their participants, or merely recruit such practitioners to their region.

Other limitations of our study include that all three studies used in our meta-analysis were conducted on residents from generalist or primary care (PC) specialties: two from Family Medicine and one from Internal Medicine. It has been previously demonstrated that participation in an IHE influences medical students towards pursuing PC careers,^20^ and these findings may not be generalizable to non-PC specialties. Additionally, two of our studies were survey-based, which risks introducing recall bias and subjectivity.^21^^,^^25^

Finally, we were unable to conduct a meta-analysis of IHEs’ impact on future practice in a LMIC, as only Bazemore et al addressed this question.^21^ They found a significant increase in such international health work among IHE participants compared to non-participants. However, they made a different discovery when they compared the practice profiles of their graduates who had completed the residency before the IHE was implemented (i.e., pre-implementation graduates) with those who completed the residency after the IHE was implemented (i.e., post-implementation graduates, including both IHE participants and non-participants). When they compared pre-implementation graduates with post-implementation graduates, they did not find any significant difference in international health work, but they did find an increase among post-implementation graduates in their work among domestic underserved populations. This may suggest that the more widespread impact of an IHE is found in future domestic rather than international service. By contrast, Russ et al found that 32 out of 94 IHE participants went on to work in a LMIC, compared to 20 participants who went on to work with domestic underserved populations.^23^

The current state of evidence regarding whether participation in IHEs will lead to increased service to underserved populations is limited by lack of rigorous study designs increasing the potential for confounding factors. First and foremost, to advance our knowledge and understanding in this area, randomised trials or carefully designed prospective non-randomised trials which account for confounding need to be designed, without information from these types of studies we are unlikely to improve the state of science on this topic. Therefore, further study of IHE participants’ international health career involvement is needed. If IHEs do not lead to increased international health engagement in the future, then one may question what benefit international host partner sites receive from their involvement in such programs. IHEs can be resource-intensive, and there is a risk of unethically burdening our often under-resourced LMIC partners if they do not benefit from the experience. Such risks and benefits have been discussed elsewhere,^26^^-^^28^ and the Working Group on Ethics Guidelines for Global Health Training (WEIGHT) provide guidelines for an ethical approach to IHE.^29^ Future study should assess the impact of applying these ethical criteria on the future careers of IHE participants. Finally, the high level of heterogeneity in our study suggests that future studies should clearly describe the IHEs included in their studies to allow for relevant sub-group analyses and sensitivity analyses. Also, the heterogeneity among studies in this field may improve with the standardization of outcomes being measured.

## Conclusion

Participation in an IHE may be effective for increasing graduates’ care for domestic underserved populations in future clinical practice. However, due to lack of randomised trials, the evidence for this effect is weak. Due to the inconsistency in the results across studies, the magnitude of the effect is unclear. Further studies using more robust methods of assessment are required to determine effectiveness and better reporting of IHEs is required for a better understanding of these programs. Likewise, further research is needed to establish whether there is an effect on future service in a LMIC setting. Given the broad range of IHEs available, both the characteristics of effective IHE models as well as potentially confounding factors in study design require further investigation.
